# Binding Stability of Antibody—α-Synuclein Complexes Predicts the Protective Efficacy of Anti-α-synuclein Antibodies

**DOI:** 10.1007/s12035-022-02824-4

**Published:** 2022-04-22

**Authors:** Matthias Höllerhage, Andreas Wolff, Tasnim Chakroun, Valentin Evsyukov, Linghan Duan, Oscar Wing-Ho Chua, Qilin Tang, Thomas Koeglsperger, Günter U. Höglinger

**Affiliations:** 1 Department of Neurology, Hannover Medical School, Hannover, D-30625 Germany; 2 Department of Neurology, Technical University of Munich (TUM), D-81675 Munich, Germany; 3 Department of Translational Neurodegeneration, German Center for Neurodegenerative Diseases (DZNE), D-81377 Munich, Germany; 4 Department of Neurology, Ludwig Maximilian University Munich, D-81377 Munich, Germany

**Keywords:** Parkinson’s disease, Alpha-synuclein, Disease models, Antibody therapy

## Abstract

Spreading of alpha-synuclein (αSyn) may play an important role in Parkinson’s disease and related synucleinopathies. Passive immunization with anti-αSyn antibodies is a promising method to slow down the spreading process and thereby the progression of synucleinopathies. Currently, it remains elusive which specific characteristics are essential to render therapeutic antibodies efficacious. Here, we established a neuronal co-culture model, in which αSyn species are being released from αSyn-overexpressing cells and induce toxicity in a priori healthy GFP-expressing cells. In this model, we investigated the protective efficacy of three anti-αSyn antibodies. Only two of these antibodies, one C-terminal and one N-terminal, protected from αSyn-induced toxicity by inhibiting the uptake of spreading-competent αSyn from the cell culture medium. Neither the binding epitope nor the affinity of the antibodies towards recombinant αSyn could explain differences in biological efficacy. However, both protective antibodies formed more stable antibody-αSyn complexes than the non-protective antibody. These findings indicate that the stability of antibody-αSyn complexes may be more important to confer protection than the binding epitope or affinity to recombinant αSyn.

## Introduction

Parkinson’s disease (PD) is the most common neurodegenerative movement disorder. Typical clinical features of PD include motor symptoms (bradykinesia, rigidity, tremor at rest, postural instability), non-motor symptoms (e.g., hyposmia, REM sleep behavioral disorder, constipation), as well as psychiatric and cognitive symptoms at later stages of the disease [1, 2]. Motor symptoms are caused by the progressive loss of dopaminergic neurons in the midbrain [3]. Affected neurons are characterized by abnormal insoluble intracellular proteinaceous inclusions, called Lewy bodies (LBs) and Lewy neurites (LNs), which are mainly composed of the protein alpha-synuclein (αSyn) [4, 5].

The progression of αSyn pathology in PD brains from one anatomical region to another in a rather stereotyped manner suggests that spreading of transmissible αSyn species occurs from diseased neurons to formerly healthy neurons [6, 7]. This hypothesis was further consolidated by the observation of “host-to-graft” transmission of αSyn pathology in human PD patients having received intrastriatal grafts of allogenic neurons [8–10]. Furthermore, a growing body of evidence from in vitro and in vivo PD models indicates that spreading-competent forms of αSyn can be released from diseased cells into the extracellular space to enter neighboring neurons and recruit endogenous αSyn to induce further aggregation [11–13]. This prion-like intercellular spreading has been proposed as major mechanism contributing to the chronic progression of PD.

Given the current lack of efficacious and approved disease-modifying therapies for synucleinopathies, there is an unmet need to develop novel therapeutics. In this regard, immunotherapy has emerged in recent years as a promising approach [14]. Active immunization, i.e., stimulating an organism’s immune systems to generate antibodies against αSyn [15], and passive immunization, i.e., administering monoclonal antibodies raised against αSyn [16], have both been used successfully to prevent propagation of αSyn pathology in different experimental models. Specifically, passive immunization has provided encouraging results, reducing αSyn aggregation and protecting neurons in vitro and in vivo in PD models [17–22]. A prior study showed that an antibody targeting C-terminal αSyn inhibited intracellular aggregation in cultured H4 neuroglioma cells [22]. Another study demonstrated the efficacy of an N-terminal anti-αSyn antibody to prevent uptake of pre-formed fibrils in cultured mouse hippocampal neurons [19]. Furthermore, passive immunization with C-terminal anti-αSyn antibodies reduced calpain-cleaved αSyn aggregates and reduced behavioral deficits and neurodegeneration in an αSyn transgenic mouse model [18, 20]. Others again showed that an N-terminal anti-αSyn antibody with high selectivity for aggregated αSyn reduced spreading of αSyn-pathology and reduced motor deficits in mice after injection of preformed αSyn fibril into the striatum [21]. Meanwhile, early clinical trials (phases I and II) have investigated the safety and disease-modifying efficacy of antibodies against αSyn in human subjects living with PD (e.g., NCT02157714, phase I; NCT02095171, phase I; NCT03716570, phase I; NCT02459886, phase I; NCT03100149, phase II; NCT03318523, phase II). Preliminary results from the PASADENA trial (NCT03100149) showed a promising trend towards slower progression in the motor examination (Unified Parkinson’s Disease Rating Scale part III) after treatment with prasinezumab, a C-terminal anti-αSyn antibody [23, 24], as compared to placebo [25–27]. On the other hand, the SPARK trial (NCT03318523), investigating the efficacy of cinpanemab, an antibody binding the N-terminus of αSyn, with high affinity towards aggregated over monomeric αSyn [21], missed the primary and secondary endpoints. In conclusion, it remains elusive which particular characteristics of anti-αSyn antibodies are essentially required to confer neuroprotection. Therefore, we aimed to explore the specific biochemical characteristics of protective vs. non-protective αSyn antibodies and their relevance for protective efficacy.

For this purpose, we established a novel co-culture system of human dopaminergic postmitotic neurons to study αSyn-spreading from αSyn-overexpressing donor cells to GFP-overexpressing recipient cells. The quantification of degeneration in recipient neurons provided evidence of αSyn-induced spreading and toxicity via the extracellular space. We then investigated three different antibodies raised against αSyn and a control antibody. Only two of the three αSyn-antibodies effectively protected neurons from toxicity induced by extracellular αSyn. We quantified their neuroprotective efficacy, their potential to deplete αSyn from the medium, their potential to block αSyn uptake into neurons, their αSyn epitope-binding domain, their specificity (off-target binding), their αSyn sensitivity (on-target binding), and the stability of antibody-αSyn complexes, aiming to identify the key determinants of protective efficacy of αSyn-targeting antibodies.

## Material and Methods

### Cell Culture

Proliferating Lund human mesencephalic (LUHMES) cells [28] were expanded in T75 flasks (EasYFlasks, Nunclon DELTA, VWR, Darmstadt, Germany) coated with 50 µg/mL poly-l-ornithine (Sigma-Aldrich, St. Louis, MO). Cells were kept in growth medium consisting of DMEM/F12 (Sigma-Aldrich), supplemented with 1% N2 supplement (Life Technologies, Carlsbad, CA) and 0.04 µg/mL basic fibroblast growth factor (bFGF; PeproTech, Rocky Hill, CT).

For differentiation, cells were seeded on T75 flasks, T25 flasks, or multi-well plates (Nunc MicroWell plates, Thermo Fisher Scientific, Waltham, MA) sequentially coated with 50 µg/mL poly-l-ornithine (Sigma-Aldrich) and 5 µg/mL bovine fibronectin (Sigma-Aldrich). Cells were cultured in differentiation medium consisting of DMEM/F12 with 1% N2 supplement, 1 µg/mL tetracycline, 0.49 µg/mL dibutyryl cyclic-AMP (Sigma-Aldrich), and 2 ng/mL glial cell–derived neurotrophic factor (GDNF; R&D Systems, Minneapolis, MN). Cells were kept in standard cell culture conditions at 37 °C, 5% CO_2_, and water-saturated air at all times. Cell density was kept at 100,000 cells/cm^2^ across all flasks and well plate formats.

### Enzyme-Linked Immunosorbent Assay

To quantify the amount of αSyn present in the cell culture medium, a solid-phase sandwich enzyme-linked immunosorbent assay (ELISA) was performed using the alpha Synuclein Human ELISA Kit (ThermoFisher Scientific, Waltham, MA, USA) according to the manufacturer’s instructions. Data were confirmed with a second human alpha synuclein ELISA kit (Abcam) according to the manufacturer’s instructions. Briefly, conditioned medium samples from untreated control and GFP- and αSyn-overexpressing were collected on days 2, 4, and 6 after transduction, and centrifuged at 2,000 × *g* for 10 min to remove cell debris. All medium samples were used undiluted, except for day 4 αSyn-overexpression medium (diluted 1:5) and day 6 αSyn-overexpression medium (diluted 1:10). The medium samples were incubated with capture and detection antibodies for 1 h, then the plate was washed and the chromogen solution was added for 15 min followed by the stop solution. Absorbance was measured with a plate reader (FLUOstar Omega, BMG Labtech, Ortenberg, Germany) at a wavelength of 450 nm. The αSyn concentrations in the medium samples were calculated from the standard curve.

### Preparation of Co-cultures of αSyn- and GFP-Expressing Cells

Cells were plated on double-coated six-well plates in differentiation medium at a density of 100,000 cells per cm^2^. Twenty-four hours after plating, the cells were transduced with adenoviral vectors expressing αSyn or green fluorescent protein (GFP) under a cytomegalovirus (CMV) promoter with a multiplicity of infections (MOI) of 2 as previously described [13, 29–31]. After another 24 h, the cell culture medium containing adenoviral vectors was removed and the cells were washed three times with phosphate-buffered saline (PBS; Life Technologies, Carlsbad, CA, USA). The cells were then incubated with Accutase (BD Biosciences, Franklin Lakes, NJ, USA) for 1 h at 37 °C and resuspended in differentiation medium. αSyn- and GFP-overexpressing cells were re-plated in 48-well plates as co-cultures of different ratios (0:100, 25:75, 50:50, 75:25, or 100:0).

### Evaluation of the Toxicity of Extracellular αSyn

Five days after re-plating of αSyn- and GFP-overexpressing cells (6 days after transduction), the cells were incubated with 1.5 µM DRAQ7 (Abcam, Cambridge, UK) for 5 min at 37 °C. DRAQ7 is an intercalating compound that is actively removed from living cells and therefore specifically stains dead cells. Thereafter, the cells were washed with PBS and fixed with a 4% paraformaldehyde solution, followed washing with PBS. One microgram per milliliter of 4′,6-diamidino-2-phenylindole (DAPI; Sigma-Aldrich) was used for nuclear counterstaining. For microscopy, images were taken with an inverted fluorescence microscope (Leica DMI 6000; Leica Microsystems, Wetzlar, Germany) equipped with an Orca-R2 camera (Hamamatsu Photonics, Hamamatsu, Japan), using the Leica Application Suite Advanced Fluorescence version 2.6 as software (Leica Microsystems). Five images per well were taken from at least five different wells per condition. The image files were blinded by renaming them using Ant Renamer version 2.12 (Antoine Potten, Brussels, Belgium) and then DAPI-positive, GFP-positive, and DRAQ7-positive cells were quantified using the cell counter plugin of the Fiji software [32]. From all cells (DAPI positive), the proportions of GFP- and DRAQ7-double-positive cells were determined.

### Treatment with Different Antibodies

The following antibodies were investigated: control antibody, EG27/1; antibodies against αSyn, 23E8, 5D12, 8A5. To investigate the protective efficacy of the individual antibodies, LUHMES cells overexpressing αSyn and GFP were co-cultured with a 50:50 ratio. Antibodies were added to the co-culture immediately after re-plating with a concentration of 25 nM and were kept in the cell culture medium until the cells were fixed and toxicity evaluated as described above.

### Immunocytochemistry and Microscopy Imaging

LUHMES neurons were plated on ibidi 8-well plates (ibidi GmbH) and treated with 25 nM of antibodies EG27/1, 23E8, 5D12, and 8A5 for 24 h. Treatments were removed and live cells were stained with cell filling dye calcein AM (Thermo Fisher Scientific), then thoroughly washed with PBS prior to fixing with 4% paraformaldehyde and 4% sucrose (Sigma-Aldrich) for 30 min at room temperature. Cells were washed three times with PBS, permeabilized with 0.1% triton X for 15 min, then washed again three times with PBS. Blocking was carried out for 30 min at room temperature with 5% donkey serum (Jackson Laboratory, Bar Harbor, ME). Fluorescently labeled secondary antibodies were incubated for 1 h at room temperature. DAPI (Invitrogen) was added to the cells in the last 10 min of secondary antibody incubation as nuclear counterstain. Cells were washed again three times with PBS and imaged with an inverted laser scanning confocal microscope (Leica SP5; Leica Biosystems, Wetzlar, Germany) using a 63 × glycerol immersion objective. Orthogonal projections were made using the Fiji software. The secondary antibody used was donkey anti mouse Alexa 594 (1:500; Invitrogen).

### Immunoprecipitation

In order to determine if the antibodies were indeed depleting αSyn from the medium, we performed an immunoprecipitation (IP). Conditioned medium was harvested on day 6 post transduction (pT6) and centrifuged at 2,000 × g for 10 min to discard cell debris then concentrated by centrifugation at 4,000 × g for 3 h at 4 °C using 3-kDa molecular weight cutoff ultrafiltration concentrators (Vivaspin 15; Sartorius, Göttingen). The BCA protein assay kit was used to determine the protein concentration, as described above. For each condition, a total protein concentration of 2 mg/mL was mixed with 100 µg of the individual antibodies (EG27/1, 23E8, 5D12, 8A5), followed by incubation overnight at 4 °C and 1-h incubation at RT. The antibodies were then captured with pre-washed protein G-coupled magnetic beads (Pierce Protein G Magnetic Beads; ThermoFisher Scientific) over a time of 1 h at room temperature. The beads with the bound antibodies were collected and washed three times with TBS-T followed by once washing with water. Thereafter, 100 µL of XT Sample Buffer (1 × ; 4 × diluted in water; Bio-Rad Laboratories) were added and samples were incubated at 95 °C for 5 min. Twenty microliters of each sample was analyzed by Western blots as described above, using the following anti-αSyn antibodies as primary antibodies: C-terminal rabbit anti-αSyn (1:500; Cell Signaling Technology), N-terminal rabbit anti-αSyn [EP1646Y] (1:500; Abcam), and an HRP-coupled anti-rabbit antibody (1:5,000, PI-1000; Vector Laboratories) as secondary antibody.

To quantify the amount of αSyn captured by the respective antibodies, the IP was performed as described above with unconcentrated conditioned medium and the antibodies were added to a final concentration of 50 nM. αSyn amounts remaining in the medium were analyzed with the alpha Synuclein Human ELISA Kit (ThermoFisher Scientific) according to the manufacturer’s instructions.

### Uptake Reduction Analysis

On day 8 of differentiation, wild-type and αSyn knockout LUHMES cells pre-treated with antibodies EG27/1, 23E8, 5D12, and 8A5 for 1 h, then conditioned medium of αSyn-overexpressing cells was added. Antibodies were applied at a final concentration of 25 nM, while the final concentration of the conditioned medium of αSyn-overexpressing cells was aimed to be equivalent to the concentration in the 50:50 co-culture condition. Cells were harvested after 6 h of coincubation with the conditioned medium, and the uptake of extracellular αSyn was evaluated by Western blot of cell homogenates. Briefly, cells were lysed in M-PER lysis buffer (Thermo Scientific Pierce Protein Biology, Waltham, MA) supplemented with protease and phosphatase inhibitor cocktail (Roche, Basel, Switzerland). Lysis consisted of a first step of incubation for 15 min on ice, followed by a freeze–thaw cycle. Cell lysates were cleared by centrifugation at 13,000 × g for 10 min at 4 °C. The cell homogenates’ concentrations were determined with the BCA protein assay kit (Thermo Scientific Pierce Protein Biology) according to the manufacturer’s instructions. Thirty-five micrograms of proteins was separated on Criterion 4–12% Bis–Tris gradient gels (Bio-Rad Laboratories) and transferred to a PVDF membrane. Membranes were probed with a rabbit monoclonal anti-αSyn primary antibody (Clone 14H2L1, 1:500; Invitrogen, Carlsbald, CA) and an HRP-coupled anti-rabbit secondary antibody (1: 5,000, PI-1000; Vector Laboratories). An HRP-coupled anti-beta actin antibody was used to control for loading of equal amounts of proteins (1:2000; Cell Signaling Technology). The HRP signal development and imaging were performed as described above.

### Dot Blots

Nitrocellulose membranes were first incubated in TBST for 4 min and then allowed to air-dry for 30 min to minimize sample diffusion. The samples were manually dotted and the membranes were air-dried for 30 min followed by incubation in 0.4% PFA (Sigma-Aldrich) for 30 min and blocking for 1 h. Primary antibodies were incubated overnight at 4 °C and anti-mouse secondary antibodies for 1 h at room temperature.

For conditioned medium and cell lysates samples, 25 µg of protein was dotted. The membranes were incubated with primary antibodies EG27/1, 23E8, 5D12, and 8A5 and an HRP-coupled anti-mouse secondary antibody (1:2500, PI-2000; Vector Laboratories). The dot blots were developed after incubation in ECL.

For affinity dot blots, the indicated amounts of recombinant human full-length αSyn (rPeptide, Bogart, GA, USA) was dotted on the nitrocellulose membranes. Primary antibodies EG27/1, 23E8, 5D12, and 8A5 were incubated overnight at 4 °C with the indicated concentrations. Thereafter, the membranes were thoroughly washed with TBST and either immediately incubated with a fluorescence coupled anti-mouse secondary (1:10,000; IRDye 800CW secondary; LI-COR Biotechnology), or incubated with a chaotropic agent ammonium thiocynate (Sigma-Aldrich) for 5 min at room temperature, or with conditioned medium overnight at 4° C, followed by incubation with a fluorescence coupled anti-mouse secondary (1:10,000; IRDye 800CW secondary; LI-COR Biotechnology). All images were obtained with an Odyssey Fc (LI-COR Biotechnology) imaging system.

### Epitope Mapping

For the epitope mapping, 1 µg of human recombinant full-length and different αSyn fragments (N-terminus, aa 1–60, rPeptide; N-terminus plus NAC (non-amyloid-component) domain, aa 1–95, rPeptide; C-terminus, aa 96–140, rPeptide, C-terminus plus NAC domain, aa 60–140, rPeptide; NAC domain, aa 60–95, JPT Peptide Technologies, Berlin, Germany) was dotted on a nitrocellulose membrane as described above. The membranes were incubated with primary antibodies EG27/1, 23E8, 5D12, and 8A5 at a concentration of 100 nM in 1 × Roti-block in TBST overnight at 4 °C, followed by 1-h incubation at RT, incubation with a HRP-conjugated secondary antibody for 1 h at RT, incubation with the ECL solution, and imaging, as described above.

### Statistical Analysis

All statistical analyses were performed using Prism 8 for Windows 64-bit, version 8.2 (GraphPad Software, San Diego, CA, USA). For multigroup comparisons, one-way analyses of variance (ANOVA) were performed. A *p*-value below 0.05 was considered statistically significant.

## Results

### αSyn-Overexpressing LUHMES Neuron Release αSyn Into the Culture Medium

**Fig. 1:**
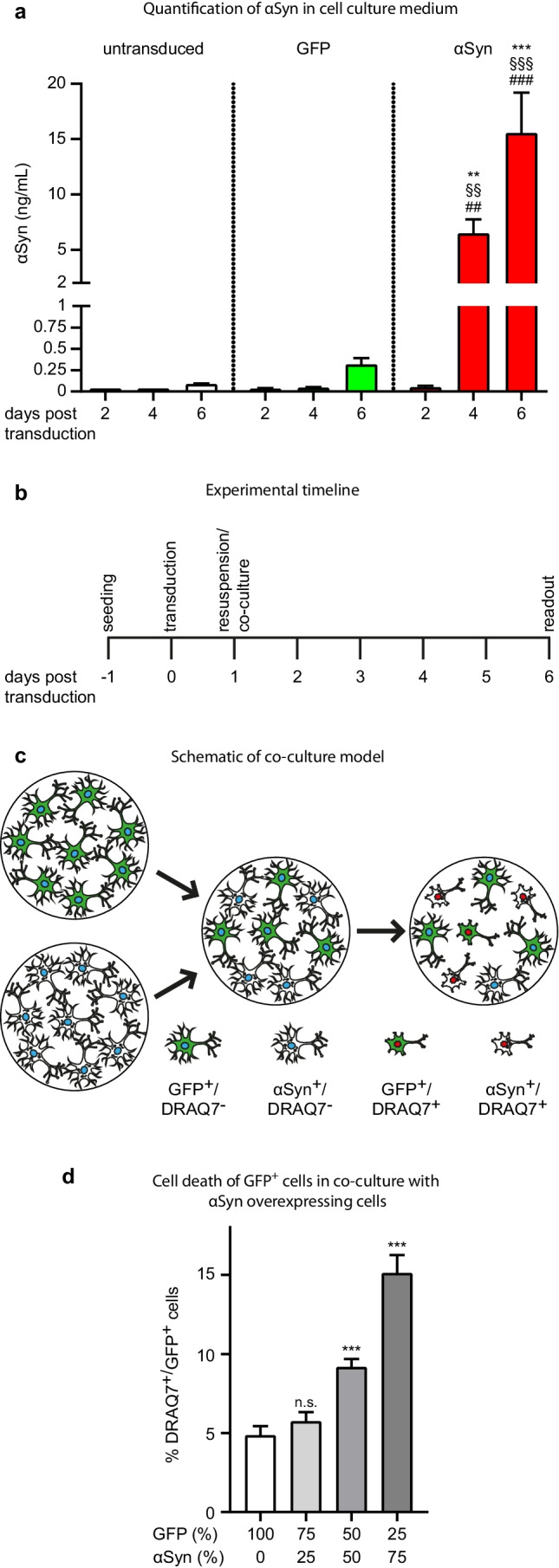
Co-culture model of GFP-expressing and αSyn-overexpressing cells. **a** In the conditioned medium of untransduced control cells (white bars) and of GFP-expressing cells (green bars), there was < 0.5 ng/ml αSyn. In cells overexpressing αSyn (red bars), on day 4 and 6 days after transduction, the αSyn concentration in the conditioned medium increased to 6.5 ± 1.2 ng/ml and 15.6 ± 3.6 ng/ml, respectively (**p < 0.01, ***p < 0.001 vs. untransduced control cells, ^§§^*p* < 0.01, ^§§§^*p* < 0.001 vs. GFP-expressing cells, ^##^*p* < 0.01, ^###^*p* < 0.001 vs. day 2 of the same condition). **b** Experimental schedule and **c** sketch of the co-culture model illustrating that cells were initially cultured and transduced separately to overexpress GFP or αSyn, respectively, then detached and replated as co-cultures. The proportion of GFP-expressing (GFP^+^) cells with DRAQ7 incorporation (DRAQ7^+^) were quantified as readout measure for cytotoxicity. **d** DRAQ7^+^ levels were low in GFP^+^ cells grown in absence of αSyn^+^ cells (white bar) and increased with increasing proportions of αSyn^+^ cells in the co-culture system (gray columns) (100% GFP-expressing cells: 4.9 ± 0.5%; 50% GFP^+^/50% αSyn^+^ cells: 9.2 ± 0.4%; 25% GFP^+^/75% αSyn^+^ cells: 15.2 ± 1.1%), n.s. not significant, ***p < 0.001 vs. GFP-expressing cells)

We have previously demonstrated that adenoviral overexpression of wild-type αSyn in human postmitotic dopaminergic LUHMES neurons is a reliable model for αSyn-induced toxicity [29, 31, 33]. We have also shown that this model results in the release of several αSyn species into the extracellular space [13]. In the current study, we performed an ELISA on the culture medium of untransduced LUHMES cells, as well as GFP- and αSyn-overexpressing cells at 2, 4, and 6 days after adenoviral transduction (days post transduction (pT): pT2, pT4, pT6) to quantify the amount of αSyn released into the extracellular medium. Barely detectable quantities of αSyn were present in the medium of untransduced control cells and GFP-transduced cells. In contrast, considerable quantities of αSyn were present in the medium of αSyn-overexpressing cells, increasing with time after transduction (Fig. 1a).

### αSyn-Overexpressing Cells Induce Degeneration of Co-cultured GFP-Expressing Cells

To establish co-cultures of GFP- and αSyn-overexpressing cells as a spreading model, we co-cultured αSyn^+^ and GFP^+^ cells (donor and recipient cells, respectively) in defined ratios (Fig. 1b–d). Toxicity in recipient cells was quantified with nuclear incorporation of the cell death marker DRAQ7 (DRAQ7^+^ as % of all GFP^+^ cells; Fig. 1c). Cultures of 100% GFP^+^ cells (0% αSyn^+^ cells) showed a low background toxicity. Cell death of GFP^+^ cells increased with increasing percentage of αSyn-overexpressing cells in the co-culture system (Fig. 1d). This indicates that degenerating αSyn-overexpressing neurons harm their healthy neighboring neurons.

### Some, but Not All Anti-αSyn Antibodies Protect Against αSyn-induced Toxicity

**Fig. 2:**
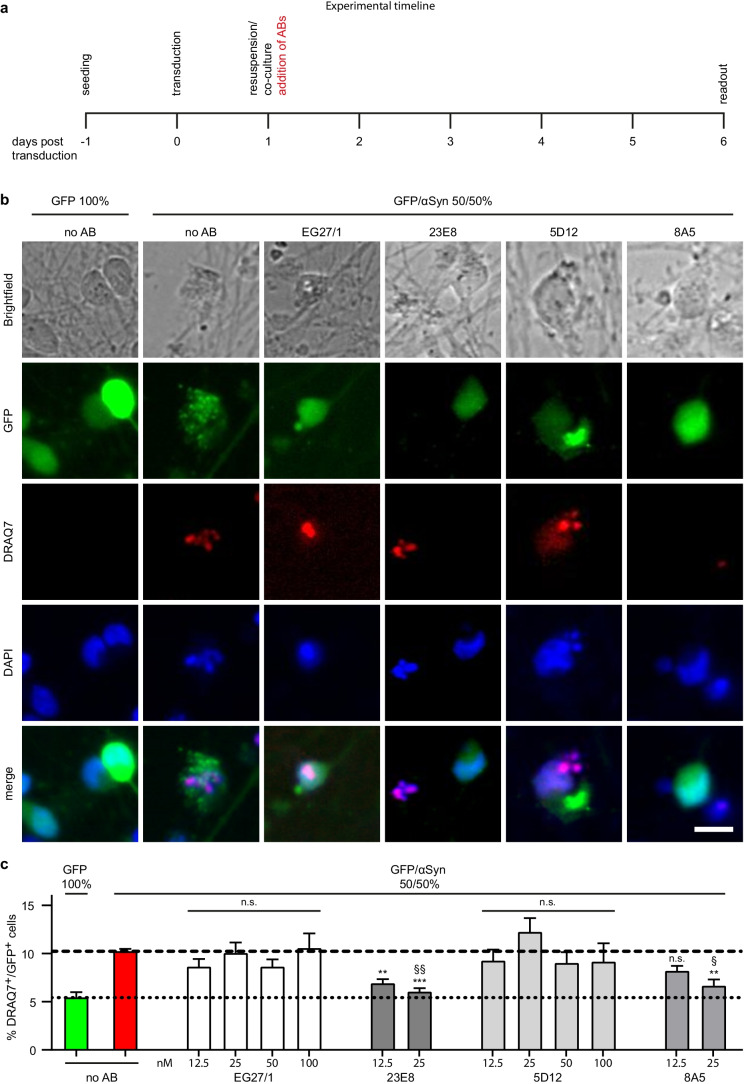
Protection of GFP-expressing cells from extracellular-αSyn-induced toxicity with anti-αSyn antibodies. **a** Experimental schedule. The antibodies were added to the culture medium on the day of preparation of the co-cultures, 1 day after adenoviral transduction to overexpress GFP or αSyn. **b** Representative microscopy images showing 100% GFP-expressing cells (left side images) or 50% GFP-expressing and 50% αSyn-overexpressing cells, respectively, either not treated with any antibody (no AB), treated with the control antibody EG27/1, or treated with one of the αSyn antibodies (23E8, 5D12, or 8A5). GFP is shown in green, DRAQ7 as marker for dying cells is shown in red, and DAPI as nuclear staining is shown in blue. The images on the bottom show merged GFP, DRAQ7, and DAPI signals. Scale bar: 10 µm. **c** Quantification of the percentage of DRAQ7^+^ among GFP^+^ cells in the corresponding culture conditions. 100% GFP^+^ cells: 5.4 ± 0.6% (green column); 50% GFP^+^/50% αSyn^+^ cells (positive control): 10.2 ± 0.3% (red column). Treatment with 12.5 nM (6.9 ± 1.2%; *p* = 0.003) and treatment with 25 nM of 23E8 (6.0 ± 0.4%; *p* < 0.001 vs positive control) and treatment with 25 nM of 8A5 (6.6 ± 0.7%; *p* = 0.001 vs positive control) significantly reduced toxicity (dark gray columns), whereas the control antibody and 5D12 were not protective in concentrations up to 100 nM. n.s. not significant, ^§^*p* < 0.05, ^§§^*p* < 0.01 vs the control antibody (EG27/1), **p < 0.01, ***p < 0.001 vs untreated 50% GFP/50% αSyn cells

To determine the protective efficacy of different antibodies raised against αSyn, 50% GFP^+^/50% αSyn^+^ cells were co-cultured and the different anti-αSyn antibodies were added to the cells 24 h after transduction (Fig. 2a). On the day of the readout (pT6), neuronal cell death in GFP^+^ cells was evaluated as described above. Baseline toxicity in cultures of 100% GFP^+^/0% αSyn^+^ cells was used as negative controls for baseline cell death. Antibody-naïve cultures of 50% GFP^+^/50% αSyn^+^ cells were considered positive controls for maximum cell death. We used increasing concentrations of each antibody starting from 12.5 nM until they showed full protection (no statistical difference in cell survival compared to untreated cells). While antibody 23E8 was protective in concentrations of 12.5 and 25 nM and antibody 8A5 was protective at 25 nM, treatment with the control antibody not binding to αSyn (EG27/1) did not reduce cell death in concentrations up to 100 nM (Fig. 2b, c; blank column). Also, the anti-αSyn antibody 5D12 did not reduce cell death in concentrations up to 100 nM (Fig. 2b, c; light gray column). However, the anti-αSyn antibodies 23E8 and 8A5 had a significant protective effect (Fig. 2b, c; dark gray columns).

### Protective Anti-αSyn Antibodies Efficiently Bind Extracellular αSyn

**Fig. 3:**
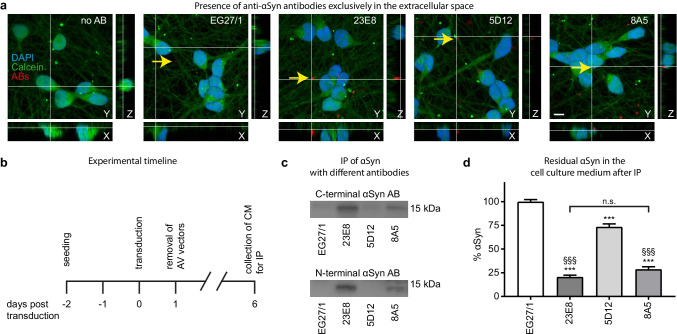
Extracellular site of action of anti-αSyn antibodies. **a** Representative confocal microcopy images of cells not treated with any antibody (no AB), treated with the control AB (EG27/1), or with one of the three anti-αSyn antibodies (23E8, 5D12, and 8A5). Nuclear DAPI staining is shown in blue, cytoplasmic calcein staining in green, and staining of the antibodies in red. Whereas all antibodies were detected in extracellular location (yellow arrows), no antibody signal was detected inside the cells, demonstrating that the antibodies did not enter the cells. Scale bar: 10 µm. **b** Experimental schedule: cells were transduced with adenoviral vectors to overexpress αSyn and conditioned medium (CM) for immunoprecipitation (IP) was collected six days post transduction. **c** Western blots of the precipitate of the CM with the control antibody (EG27/1) or the three anti-αSyn antibodies (23E8, 5D12, and 8A5), showing that clearly 23E8 and 8A5, less so 5D12, but not but EG27/1 eluted αSyn (top panel: immunostaining with a C-terminal αSyn antibody; bottom panel: immunostaining with an N-terminal αSyn antibody). **d** Quantification of residual αSyn in the CM after immunoprecipitation by ELISA shows that all anti-αSyn antibodies led to a significant reduction of αSyn compared to the control antibody. The non-protective anti-αSyn antibody 5D12, however, only reduced αSyn levels by 26.6 ± 3.1%, whereas the protective anti-αSyn antibodies reduced αSyn by 79.5 ± 1.9% (23E8) and 71.4 ± 2.7% (8A5), compared to the control antibody. n.s. not significant ***p < 0.001 vs. the control antibody (EG27/1). ^§§§^*p* < 0.001 vs. the non-protective anti-αSyn antibody (5D12)

To understand the mode of action of the protective antibodies, we first investigated the localization of all antibodies 24 h after addition to LUHMES cells. Alexa 594-coupled anti-mouse IgG secondary antibodies were used to visualize the localization of the primary antibodies. Cells were prefilled with calcein as an intracellular counterstain, and DAPI was used as a nuclear stain. Orthogonal projections of confocal z-stacks were acquired (Fig. 3a). None of the tested antibodies entered into the cytoplasm of cultured cells. We could not detect any signal corresponding to anti-αSyn antibodies in the intracellular space, indicating that their biological activity takes place in the extracellular space.

Next, we investigated the extracellular αSyn-binding capacities of the different antibodies. As shown schematically in Fig. 3b, conditioned medium from αSyn-overexpressing cells 6 days post transduction was collected and immunoprecipitated using the control antibody EG27/1 and the anti-αSyn antibodies (23E8, 5D12, 8A5). Precipitated αSyn was evaluated by Western blot (Fig. 3c). The control antibody EG27/1 did not extract detectable quantities of αSyn from the conditioned medium, the anti-αSyn antibody 5D12 only extracted very little αSyn, whereas the two protective anti-αSyn antibodies 23E8 and 8A5 extracted significantly higher levels of αSyn (Fig. 3c). Quantification of αSyn remaining in the conditioned medium after immunoprecipitation with ELISA showed that the αSyn-binding antibodies 23E8 and 8A5 were able to deplete 79.5 ± 1.9% and 71.4 ± 2.7% of total αSyn from the conditioned medium, compared to the control antibody, whereas the αSyn-binding antibody 5D12 depleted only 26.6 ± 3.1% (Fig. 3d).

Together, these findings indicate that protective anti-αSyn antibodies 23E8 and 8A5 have an extracellular mode of action that involves the efficient binding of large quantities of extracellular αSyn.

### Protective Anti-αSyn Antibodies Reduce αSyn Uptake Into LUHMES Neurons

**Fig. 4:**
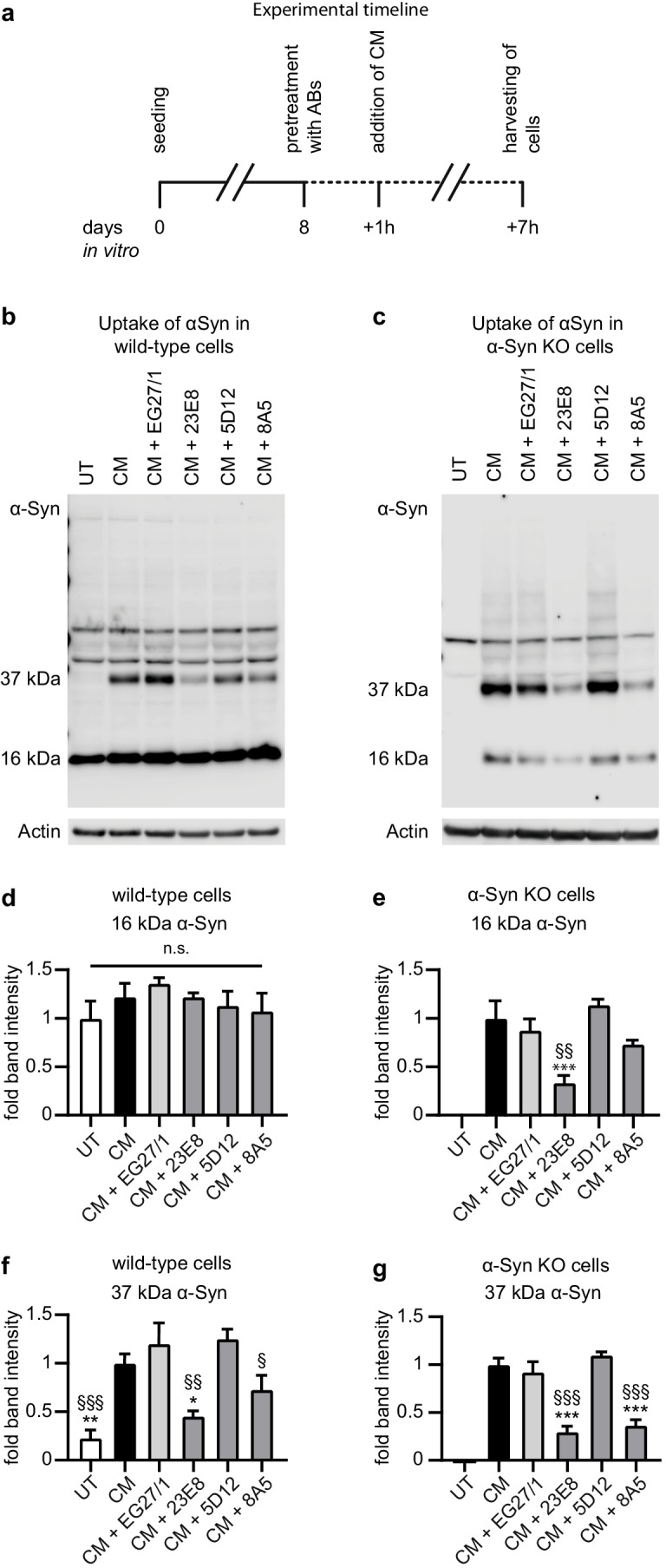
Reduction of mainly oligomeric intracellular αSyn by anti-αSyn antibodies. **a** Experimental schedule: cells were differentiated for 8 days, preincubated with the antibodies for 1 h and then treated with conditioned medium (CM) from αSyn-overexpressing cells for 6 h, before harvesting. **b** Western blot of cell lysates from untreated wild-type LUHMES cells (UT), wild-type LUHMES cells treated with CM from αSyn-overexpressing LUHMES cells (CM) without or with the control antibody (EG27/1) or an anti-αSyn antibodies (23E8, 5D12, 8A5). Treatment with CM from αSyn-overexpressing cells led to the occurrence of a specific 37 kDa oligomeric αSyn band. **c** Western blot of cell lysate from αSyn knockout cells, untreated or treated with CM as described in **b**. Expectedly in untreated αSyn knockout cells, there was no αSyn present, whereas treatment with CM led to the occurrence of monomeric and more dominantly oligomeric αSyn. **d** Quantification of monomeric αSyn showed no differences between the experimental conditions in wild-type cells. **e** Quantification of monomeric αSyn in knockout cells showed that treatment with anti-αSyn antibody 23E8 led to a significant reduction of monomeric αSyn present after treatment with CM. Treatment with anti-αSyn 8A5 showed a clear trend towards a reduction of monomeric αSyn, whereas treatment with the non-protective anti-αSyn antibody 5D12 did not reduce uptake of monomeric αSyn. **f** Quantification of 37-kDa oligomeric αSyn occurring after treatment of wild-type LUHMES cells with CM from αSyn-overexpressing cells. Treatment with antibodies 23E8 and 8A5 led to a reduction of oligomeric αSyn compared to the control antibody (EG27/1). **g** Quantification of 37-kDa oligomeric αSyn in αSyn knockout cells after treatment with CM. Treatment with 23E8 and 8A5 led to a reduction of oligomeric αSyn, occurring after treatment with CM, also in knockout cell. n.s. not significant, *p < 0.05, **p < 0.01, ***p < 0.001 vs CM; ^§^*p* < 0.05, ^§§^*p* < 0.01, ^§§§^*p* < 0.001 vs control antibody EG27/1

Next, we studied if the protective efficacy of the anti-αSyn antibodies 23E8 and 8A5 would result from blocking the uptake of extracellular αSyn into LUHMES neurons. Therefore, LUHMES cells were differentiated for 8 days and then treated with the antibodies for 1 h prior to addition of conditioned medium from αSyn-overexpressing cells. Six hours later, the cells were harvested (Fig. 4a) and the presence of αSyn in the cell lysates was then evaluated by Western blot (Fig. 4b, c). Untreated cells were used as control for the baseline level of cell-endogenous αSyn. Cells treated with conditioned medium without the addition of antibodies were used as the positive control for the uptake of extracellular αSyn. Both wild-type (WT) and αSyn-knockout (KO) LUHMES cells (previously described in [13]) were used as recipient cells to analyze the uptake of both monomeric and oligomeric αSyn (observed at 16 kDa and 37 kDa, respectively [13]; Fig. 4b, c).

Uptake of monomeric αSyn could not be detected in WT cells because of the high endogenous αSyn levels (Fig. 4b, d). In KO cells, however, it became obvious that monomeric αSyn was indeed taken up from the conditioned medium (Fig. 4c, e). The levels of monomers taken up from conditioned medium were markedly reduced in cells that were pretreated with the protective antibody 23E8 (62.0 ± 8.5% reduction; *p* = 0.0004, Fig. 4e) and showed a trend in cells that were pretreated with the protective antibody 8A5 (16.6 ± 4.9% reduction; *p* = 0.31, Fig. 4e) compared to cells exposed to conditioned medium without antibody pretreatment. Pretreatment with the control antibody EG27/1 and non-protective anti-αSyn antibody 5D12 did not significantly reduce the levels of intracellular monomeric αSyn taken up from conditioned medium (Fig. 4e).

Treatment with conditioned medium also led to the appearance of an intracellular oligomeric αSyn species at ~ 37 kDa in both WT and KO cells (Fig. 4b, c). In comparison to the control antibody, pretreatment with the protective antibodies 23E8 and 8A5 strongly reduced this band in WT cells (23E8: 62.3 ± 4.5% reduction, *p* = 0.001; 8A5: 39.2 ± 12.1% reduction, *p* = 0.02; Fig. 4f) and in αSyn-KO cells 23E8: 67.7 ± 6.4% reduction, *p* < 0.001, 8A5: 60.3 ± 6.2%, *p* < 0.001; Fig. 4g). Pretreatment with the control antibody EG27/1 and non-protective anti-αSyn antibody 5D12 did not significantly reduce the levels of intracellular oligomeric αSyn taken up from conditioned medium in WT and in αSyn-KO cells (Fig. 4f, g). From these results, we concluded that protective antibodies, but not the non-protective antibodies, prevented the uptake of spreading-competent αSyn from the extracellular space and consistently reduced the levels of intracellular oligomeric αSyn.

### Specific Epitope Recognition Does Not Explain the Protective Properties of 23E8 and 8A5

**Fig. 5:**
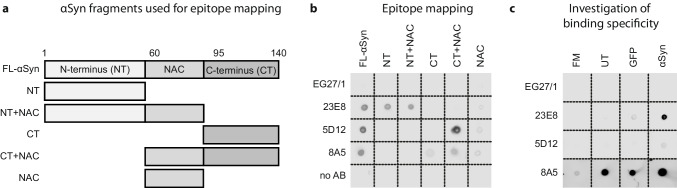
Analysis of the binding properties of the different antibodies. **a** Schematic illustration of monomeric full-length αSyn (FL-αSyn) and different recombinant αSyn fragments consisting of the N-terminus (NT), the C-terminus (CT), and/or the NAC-domain, used to investigate the binding site of the different antibodies. **b** Dot blot analysis showing the binding of the four antibodies against the different forms of αSyn illustrated in **a**. Expectedly, the control antibody (EG27/1) did not bind any form of αSyn. Anti-αSyn antibody 23E8 bound to both fragments containing the N-terminus, whereas anti-αSyn antibodies 5D12 and 8A5 bound to C-terminal fragments. With the antibodies omitted (no AB) there was no signal. **c** Dot blot investigating the binding of antibodies to fresh medium (FM), and conditioned medium from untransduced cells (UT), from GFP-expressing cells (GFP), and from αSyn-overexpressing cells (αSyn). The control antibody did not bind to any medium. Anti-αSyn antibody 23E8 showed strong binding only to CM from αSyn-overexpressing cells. Anti-αSyn antibody 5D12 showed only very little binding to CM from αSyn-overexpressing cells, whereas anti-αSyn antibody 8A5 showed strong binding to all conditioned media and little binding to fresh medium

To understand why the αSyn-binding antibodies 23E8 and 8A5, but not the αSyn-binding antibody 5D12, reduced the levels of intracellular oligomeric αSyn and protected from αSyn-induced neurodegeneration, we next explored whether the recognition of specific αSyn epitopes would be sufficient to explain these differences. Therefore, we determine the binding sites of the anti-αSyn antibodies using recombinant full-length αSyn and defined αSyn fragments (depicted in Fig. 5a).

Expectedly, the control antibody (EG27/1) did not bind to any form of αSyn, and all anti-αSyn antibodies recognized full-length αSyn. 23E8 detected both N-terminal fragments. Both antibodies 5D12 and 8A5 detected the C-terminal fragments (Fig. 5b).

Since the two protective anti-αSyn antibodies (23E8 and 8A5) bound to different domains (N-terminus and C-terminus, respectively), we concluded that recognition of a specific epitope is not sufficient to explain differences in the protective efficacy.

### Specificity Towards αSyn Alone Does Not Explain the Protective Properties of 23E8 and 8A5

In order to test for specificity of the antibodies, we investigated the binding of the different antibodies to fresh medium and conditioned media from untransduced cells, GFP-expressing cells, and αSyn-overexpressing cells (Fig. 5c). The control antibody EG27/1 did not bind to any medium. The non-protective anti-αSyn antibody 5D12 showed only very weak binding to conditioned medium from αSyn-overexpressing cells, but not to other media. The protective anti-αSyn antibody 23E8 showed strong binding to conditioned medium from αSyn-overexpressing cells, weak binding to medium from GFP cells, but not to other media. The protective anti-αSyn antibody 8A5 showed strong binding to conditioned medium from αSyn-overexpressing cells, from untransduced cells, and GFP-expressing cells, but not unconditioned control medium (Fig. 5c).

Since high quantities of αSyn were only present in the conditioned medium from αSyn-overexpressing cells (Fig. 1a), these data confirm a higher sensitivity of the protective anti-αSyn antibodies 8A5 and 23E8 over the non-protective anti-αSyn antibody 5D12 and imply a higher specificity of 23E8 compared to 8A5. These data suggest that the absence of unspecific binding (i.e., high specificity) was not an essential prerequisite of anti-αSyn antibodies to confer protection in our model.

### Sensitivity Towards αSyn Alone Does Not Explain the Protective Properties of 23E8 and 8A5

Next, we investigated if quantitative differences in sensitivity towards recombinant full-length αSyn could explain the different biological efficacies of anti-αSyn antibodies.

**Fig. 6:**
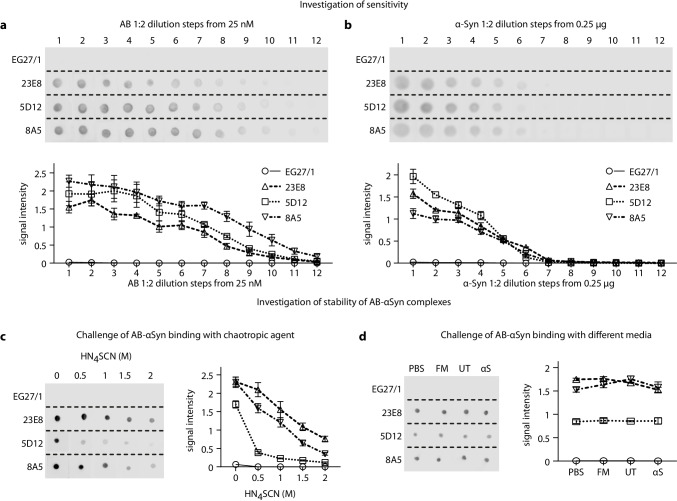
Analysis of sensitivity of the antibodies and the stability of the αSyn-antibody complexes. **a** Investigation of the sensitivity towards recombinant monomeric αSyn in a stepwise 1:2 dilution series starting with a 25 nM antibody solution and a constant αSyn quantity. The upper panel shows the dot blots. The lower panel shows the corresponding quantification. The non-protective anti-αSyn antibody 5D12 showed a sensitivity towards recombinant αSyn of intermediate level, lying between the protective anti-αSyn antibodies 23E8 and 8A5. Expectedly, the control antibody EG27 showed no sensitivity towards recombinant αSyn. **b** Investigation of the sensitivity towards recombinant monomeric αSyn in a stepwise 1:2 dilution series starting with 0.25 µg αSyn and a constant antibody concentration of 25 nM. The lower panel shows the quantification. The sensitivity towards recombinant αSyn was highest with the non-protective anti-αSyn antibody 5D12. **c** Investigation of the stability of αSyn-antibody complexes after challenging with different concentrations of the chaotropic compound ammonium thiocyanate (HN_4_SCN), showing that 23E8-αSyn and 8A5-αSyn complexes were more stable that 5D12-αSyn complexes. The symbols for the different antibodies are the same as in **a** and **b**. **d** Investigation of the stability of αSyn-antibody complexes after challenge with PBS, fresh medium (FM), conditioned medium from untransduced cells (UT), and conditioned medium from αSyn-overexpressing cells (αS), showing again that 23E8-αSyn and 8A5-αSyn complexes were more stable that 5D12-αSyn complexes. The comparison of the signals obtained from each antibody between challenging with PBS or the different media revealed no significant differences within each antibody. The symbols for the different antibodies are the same as in **a** and **b**

First, we incubated a constant concentration of recombinant αSyn with gradually decreasing concentration of each antibody in a dot blot analysis and quantified the resulting optical density (Fig. 6a). Interestingly, in this experiment, the non-protective antibody anti-αSyn antibody 5D12 showed a sensitivity to recombinant αSyn between the two protective anti-αSyn antibodies (23E8, 8A5).

Then, we incubated gradually decreasing concentrations of recombinant αSyn with a constant concentration of each antibody (Fig. 6b). In this experimental setting, the non-protective anti-αSyn antibody even showed a higher sensitivity than the two protective antibodies (23E8, 8A5).

Expectedly, the control antibody (EG27/1) did not bind recombinant αSyn (Fig. 6a, b). These data indicate that sensitivity towards recombinant αSyn could not explain the differences in protection efficacy of the anti-αSyn antibodies. Furthermore, these data show that binding to recombinant αSyn (Fig. 6a, b) does not necessarily reflect the binding to αSyn from a biological sample, e.g., in our case from conditioned medium (Fig. 5c).

### The Stability of the Antigen–Antibody Complexes Explains the Protective Properties of 23E8 and 8A5

In addition to specificity and sensitivity, stability of antibody-antigen complex or resilience against disruptive factors in more complex models is an important characteristic of antibodies. One established method to estimate the stability of the antigen–antibody complex is by semi-quantitatively measuring the resistance against a chaotropic agent [34].

We found that antibody-αSyn complexes formed by the two protective anti-αSyn antibodies (23E8, 8A5) were much more stable than complexes formed by the non-protective anti-αSyn antibody 5D12 (Fig. 6c). Our findings indicate that the complex stability of both protective anti-αSyn antibodies 23E8 and 8A5 to αSyn was approximately three times as high as the complex stability of the non-protective antibody 5D12 to αSyn (Fig. 6c, right panel). We further questioned whether these antigen–antibody complexes were differently affected in a biological context where the extracellular medium contains a complex mixture of proteins (e.g., growth factors), which could compete for binding capacities. Therefore, instead of a chaotropic agent, we used PBS, fresh medium, conditioned medium from untransduced cells, and conditioned medium from αSyn-overexpressing cells to challenge the complexes formed between recombinant αSyn and the different antibodies (Fig. 6d). We did not observe any differences in the signals between PBS and the different media, indicating that they contained no competing elements to hinder the antibodies’ binding to αSyn. The non-protective anti-αSyn antibody 5D12, however, yielded a much lower signal than the two protective anti-αSyn antibodies 23E8 and 8A5 across all conditions, showing again that the non-protective antibody had a lower complex stability with αSyn than the two protective antibodies.

Together, these data indicate that two protective anti-αSyn antibodies 23E8 and 8A5 are much less likely to dissociate from αSyn compared to 5D12. This would contribute to explaining their protective properties against αSyn-mediated toxicity (Fig. 2) and their efficacies in eluting extracellular αSyn from the medium (Fig. 3).

## Discussion

Spreading of αSyn species from cell to cell is believed to be a major contributor to the propagation of αSyn pathology throughout the brain in patients suffering from PD and other synucleinopathies [12, 35]. Therefore, passive immunization strategies to scavenge harmful αSyn species and thereby preventing cell-to-cell spreading are being developed as experimental disease-modifying therapies. Nonetheless, the exact αSyn species involved in cell-to-cell spreading is still under debate. In order to design antibodies that are effective in preventing cell-to-cell spreading, a better understanding of the spreading species and essential biochemical properties of therapeutic anti-αSyn antibodies is very important.

In order to investigate different anti-αSyn antibodies, we first established a novel cell model of cell-to-cell spreading of αSyn, in which GFP-expressing recipient cells were co-cultured with αSyn-overexpressing cells. Previously, we showed that overexpression of αSyn can be used as a model to study direct toxic effects of αSyn within LUHMES cells [29–31, 33]. Furthermore, we showed that distinct αSyn species are taken up by LUHMES cells and lead to intracellular aggregation and toxicity [13]. Moreover, preformed fibrils of αSyn are used to model aggregation induced by extracellular αSyn [36]. The main advantage of the experimental setup used in the present study is that it utilizes cell-derived αSyn instead of exogenous αSyn as spreading species. Furthermore, the recipient cells express GFP and are therefore readily identifiable by fluorescence microscopy. Therefore, this co-culture model is a valuable tool to investigate various intervention strategies to reduce intercellular spreading of αSyn. Furthermore, previous studies used neuroblastoma cell lines or mouse primary neurons to investigate potentially neuroprotective anti-αSyn antibodies [19, 22], whereas LUHMES cells, used in this model, have the advantage that they are directly derived from human midbrain neurons and resemble human postmitotic dopaminergic cells of the substantia nigra very closely [28, 33, 37], the demise of which is responsible for motor symptoms in PD [38].

Several lines of investigation support cell-to-cell transmission of various αSyn species as an underlying mechanism of pathology propagation in synucleinopathies [39–42]. Herein, we further support the prior evidence by showing that cells that suffer from αSyn pathology negatively impact the survival of their neighboring cells. As we know from our prior studies, αSyn-overexpressing cells exhibit approx. 50% toxicity levels at the studied time point [29, 33], which might lead to an overall hostile environment for the recipient cells. However, while we cannot be certain that cell-derived αSyn alone was responsible for the observed toxicity in GFP cells, it is safe to assume that it is at least responsible for a significant part of it since it could be reduced by specific anti-αSyn antibodies 23E8 and 8A5. Moreover, in patients’ brain, αSyn involved in disease propagation might also result from release mechanisms as well as dying cells [43].

It is also worth mentioning that the αSyn concentration we measured in the conditioned medium is in the range of the concentration of 0.5 to 8 ng/ml measured in human brains [44]. Additionally, the antibody concentration we used (25 nM, ~ 3.75 µg/ml) was very close to what can be achieved in human brains, given that ~ 0.4% of systemically administered αSyn antibodies cross the blood–brain barrier [45–47]. In the PASADENA clinical trial (NCT03100149), in which antibodies against αSyn were investigated, 6 g of antibody was administered [23], corresponding to 1.2 mg/ml at blood volume of 5 l or 4.8 µg/ml (0.4%) in the intrathecal compartment.

Furthermore, the two protective anti-αSyn antibodies were able to bind and significantly reduce extracellular αSyn, and to efficiently decrease the amount of αSyn taken up by naïve cells. Together with the observation that these antibodies remain exclusively in the extracellular space, we concluded that their specific mode of action relies on extracellular scavenging of αSyn. In line with our conclusions, several studies demonstrated blocking of uptake of exogenous pathological species of αSyn and tau as a prominent mechanism of immunotherapies [19, 48].

The exact release and uptake mechanisms of monomeric and oligomeric αSyn are not yet fully understood. It was however previously shown that monomeric, but not aggregated, αSyn is able to pass the cell membrane boundaries through diffusion [49]. In the context of this study, we could assume that both monomeric and oligomeric αSyn were passively released from αSyn-overexpressing cells since the integrity of the cell membrane is compromised as a result of toxicity. In addition, we observed that both monomeric and oligomeric αSyn were taken up by naïve cells within a relatively short time frame (6 h). Even though monomeric αSyn can be taken up by either passive or active mechanisms, previous studies suggest that oligomeric αSyn can only be taken up through active mechanisms [43]. A passive uptake of monomers and an active uptake of oligomers might explain why the uptake of oligomeric species seemed to prevail over monomeric αSyn in our experiments, as shown by more prominent oligomeric αSyn bands compared to monomer bands after uptake of αSyn from the medium in αSyn KO (Fig. 4c).

Furthermore, the presence of oligomeric αSyn species in recipient cells suggests their uptake from the conditioned medium as such. Accordingly, we previously showed that oligomeric αSyn species are already present in the conditioned medium in αSyn-overexpressing LUHMES cells [13]. Alternatively, the oligomeric αSyn band occurring after uptake of αSyn could also result from aggregation of monomeric αSyn in the intracellular space after uptake. The latter seems, however, less likely since we previously showed that the intracellular αSyn knockout environment is not favorable to aggregation due to the lack of endogenous αSyn as a substrate [13]. Strikingly, even though the protective anti-αSyn antibodies 23E8 and 8A5 reduced the uptake of total αSyn in recipient cells, the reduction of oligomeric αSyn appeared to be more prominent than that of monomers (Fig. 4e, g). Based on our current results, we cannot conclude whether monomeric αSyn, oligomeric αSyn, or the combination of both was responsible for toxicity in recipient cells. However, we previously showed a positive correlation between cell death in LUHMES cells and the quantities of this particular oligomeric species (appearing at approx. 37 kDa) present in the intracellular space and toxicity [29, 33]. In addition, other groups also observed toxic effects of αSyn oligomers of a similar size [50, 51]. In light of this, it might be considered that this particular aggregated form of αSyn is responsible for toxicity and that its specific capture by antibodies the protective anti-αSyn 23E8 and 8A5 confers their protective efficacy.

To explain the differences in biological efficacy between protective and non-protective antibodies, we followed different approaches to determine the specific characteristics of the antibodies. Previous studies suggested that binding to distinct αSyn epitopes is an important property of αSyn antibodies. However, the results have been inconclusive so far. For instance, it was shown that binding to a distinct epitope in the C-terminus of αSyn might be essential by inhibiting αSyn cleavage via calpain-1 in an αSyn mouse model [20]. Others found that C-terminal antibodies could be effective regardless of the calpain-1 binding site in transgenic mice [18]. There are also other reports showing effectiveness of N-terminal antibodies in a transgenic A53T-αSyn mouse model [21]. Furthermore, also in clinical trials, C-terminal (NCT03100149) and N-terminal (NCT03318523) antibodies were investigated in patients, showing that presently there is no consensus yet about the best target epitope of αSyn for passive immunization approaches.

In line with that, we also found that one antibody targeting an N-terminal epitope (23E8) and one antibody targeting a C-terminal epitope (8A5) had comparable protective capabilities. However, one C-terminal antibody (5D12) displayed no protection potential, both in our model and in an αSyn transgenic mouse model [20]. Together, these findings suggest that targeting one specific region of αSyn is not sufficient to explain protective efficacy of therapeutic antibodies, and that other properties must be important.

We also found that one of the two protective anti-αSyn antibodies (23E8) only bound protein in conditioned medium from αSyn-overexpressing cells, whereas the other protective anti-αSyn antibody (8A5) bound also to protein from conditioned medium of untransduced and GFP-overexpressing cells, suggesting a lower specificity of 8A5 compared to 23E8. Since the antibodies had similar protective efficacies, our data suggest that high specificity is not a mandatory characteristic of efficacious anti-αSyn antibodies to confer protection in our model. However, lack of specificity might be of greater relevance in vivo resulting in increased off-target binding and more adverse effects [52].

When investigating the sensitivity of the different antibodies to recombinant αSyn, we did not observe differences between protective and non-protective antibodies, suggesting that sensitivity to recombinant αSyn per se could not predict the antibodies’ behavior in biological systems. However, by challenging the stability of antibody-antigen complexes with a chaotropic agent (ammonium thiocyanate), we showed that the two protective antibodies formed more stable complexes with αSyn in comparison to the non-protective antibody. Indeed, the stability of antigen–antibody complexes to a chaotropic agent directly correlates to the affinity index of such complexes [34], suggesting that the protective antibodies had higher affinity indexes than the non-protective antibody. Furthermore, the complexes formed between αSyn and non-protective antibody 5D12 were also less stable when challenged with PBS or medium. However, all αSyn-specific tool antibodies displayed comparable sensitivity and therefore most likely also comparable affinity in a binary antigen–antibody setting. Differences in affinity index between protective and non-protective antibodies indicate differences in overall stability of the complexes and resistance against chaotropic agents and other interfering factors. Therefore, we propose that the stability of the complexes between αSyn and therapeutic antibodies is detrimental for the protective efficacy. Despite the observation that the protective anti-αSyn antibody 8A5 appeared to be less specific than 23E8, challenging of antibody-antigen complex with conditioned medium did not result in disruption of complexes formed between 8A5 and αSyn, suggesting that a strong resilience against disruptive factors can compensate for reduced specificity.

## Conclusion

In conclusion, we tested three different potentially therapeutic anti-αSyn antibodies in a novel co-culture model of cell-to-cell spreading of pathological αSyn species. Two out of three anti-αSyn antibodies were able to significantly reduce toxicity in recipient cells. The antibodies seemed to be acting exclusively by binding αSyn species in the extracellular space and thereby preventing their uptake into neighboring healthy cells. Most importantly, we found that the binding epitope, sensitivity towards recombinant αSyn, and specificity were not the most relevant characteristics to confer protective efficacy for therapeutic antibodies. Instead, a good sensitivity towards αSyn species in the relevant biological sample and high stability of complexes with αSyn (i.e., formation of stable complexes resistant to dissociation) were more important. Our data underline the need to test anti-αSyn antibodies in biological system that reflect the spreading αSyn-species in patients as accurately as possible.

## Data Availability

The datasets used and/or analyzed during the current study are available from the corresponding author on reasonable request.
